# 

**Published:** 1881-09

**Authors:** 


### CHICAGO MEDICAL Journal and Examiner

**EDITORS:**

N. S. DAVIS, M.D., LL.D. JAS. NEVINS HYDE, A.M., M.D.

DANIEL R. BROWER, A.M. M.D.

CHICAGO MEDICAL PRESS ASSOCIATION. AUG. 2D 1875.

CHICAGO:

*PUBLISHED BY THE CHICAGO MEDICAL PRESS ASSOCIATION,*

188 CLARK STREET.

☞ Read the Notice of this Journal among Advertisements.

